# Progress in the application of novel inflammatory indicators in chronic kidney disease

**DOI:** 10.3389/fmed.2025.1500166

**Published:** 2025-01-30

**Authors:** Wenrui Gao, Xiangyu Wang, Yulin Zou, Sheng Wang, Jun Dou, Senlin Qian

**Affiliations:** ^1^Department of Nephrology, The Third Clinical Medical College of China Three Gorges University, Gezhouba Central Hospital of Sinopharm, Yichang, Hubei, China; ^2^School of Basic Medicine, China Three Gorges University, Yichang, Hubei, China; ^3^Department of Neurology, The Third Clinical Medical College of China Three Gorges University, Gezhouba Central Hospital of Sinopharm, Yichang, Hubei, China; ^4^Department of Geriatrics, The Third Clinical Medical College of China Three Gorges University, Gezhouba Central Hospital of Sinopharm, Yichang, Hubei, China; ^5^Department of Clinical laboratory, The Third Clinical Medical College of China Three Gorges University, Gezhouba Central Hospital of Sinopharm, Yichang, Hubei, China

**Keywords:** chronic kidney disease, micro-inflammation, neutrophil-to-lymphocyte ratio, platelet-to-lymphocyte ratio, systemic immune-inflammation index, relationship, prognosis

## Abstract

Chronic kidney disease has become a public health problem endangering the health of all humans because of its high prevalence, high mortality and high medical burden. The chronic micro-inflammatory state is recognized as a significant component of CKD, playing a key role in disease progression. Intervening in chronic inflammation during the disease course can enhance prognosis. Recent studies have demonstrated that novel inflammatory indices, such as the neutrophil-to-lymphocyte ratio, platelet-to-lymphocyte ratio, and systemic immune-inflammatory index are closely associated with CKD, meanwhile may serve as prognostic monitors of all-cause death and poor renal prognosis for the disease. This article comprehensively reports on the mechanisms of micro-inflammation in CKD, the relationship between inflammatory indicators and CKD, and their impact on prognosis.

## 1 Introduction

Chronic kidney disease (CKD) is a range of chronic conditions that affect the structure and function of the kidneys (1). The prevalence of CKD is rising globally, driven by significant increases in diseases such as diabetes, hypertension, and obesity, coupled with an aging population (2). It is reported that the global prevalence rate of CKD is more than 10%, and the disease causes nearly 10 million deaths every year (3, 4). CKD not only has a serious impact on individuals and their families, but also imposes a huge global economic burden, especially in many developing countries, where the annual cost of kidney replacement therapy often exceeds gross national income per capita (5). It is estimated that about 100 million people are pushed into poverty each year due to high medical costs, and CKD is considered to be one of the leading causes of household poverty worldwide (6). In addition, patients with CKD often suffer from mental health problems such as anxiety and depression due to physical limitations, discomfort caused by the disease, heavy financial pressure, and concern about the prognosis of the disease, which not only increase the frequency of multiple hospitalizations and increase medical expenses, but also significantly affect the overall prognosis of patients (7). Therefore, CKD has become a public health problem that endangers the health of all humans.

Chronic micro-inflammatory is recognized as a critical component of CKD, playing a pivotal role in its development by directly damaging kidney structures, disrupting physiological functions, and heightening the risk of complications (8). Interventions targeting chronic inflammation are anticipated to enhance the quality of life and reduce mortality among CKD patients (9). Therefore, investigating inflammatory markers associated with CKD disease is of paramount importance for its diagnosis and treatment. Currently, the main indicators used to evaluate the micro-inflammatory status of patients with CKD include hypersensitive C-reactive protein (hs-CRP), interleukin-1 (IL-1), interleukin-6 (IL-6) and tumor necrosis facto *α* (TNF-α) (10). However, because these indicators are not routine detection items, and some of them are limited by detection technology and cost, their application has not been widely popularized in clinical practice, especially in primary medical institutions. In contrast, a series of novel inflammatory indicators derived from blood routine, such as neutrophil to lymphocyte ratio (NLR), platelet-lymphocyte ratio (PLR), and systemic immunoinflammatory index (SII), showed significant advantages. These indicators are calculated using only neutrophil, platelet, and lymphocyte data already available in routine blood tests at no additional cost, making them especially cost-effective and practical in resource-limited Settings. In addition, compared with the simple cells count, these composite indicators can more stable and sensitive reflect the chronic inflammatory state of the body (11). In recent years, such indicators have been widely used to monitor the prognosis of various systemic diseases (12–15). Numbers studies (16–23) have highlighted the close relationship between these inflammatory indicators and CKD, which can not only predict the occurrence of various complications of CKD, but also have predictive value in the prognosis of all-cause death and adverse renal outcomes. This article comprehensively reports on the mechanisms of micro-inflammation in CKD, the relationship between inflammatory indicators and CKD, and their impact on prognosis, with the aim of providing new insights for the clinical management of CKD.

## 2 Mechanism of micro-inflammation in CKD

The micro-inflammatory status in CKD indicates that the patient exhibits no significant clinical manifestations related to infection. However, inflammatory proteins and inflammatory factors in the bloodstream, such as hs-CRP, TNF-*α*, IL-1, and IL-6, demonstrate a sustained mild increase. This phenomenon is not due to infection but rather an immune-mediated inflammatory response (24), which involves various factors and pathways.

### 2.1 Toxin accumulation and micro-ecological dysregulation

As the glomerular filtration rate gradually declines, various toxins and metabolites accumulate in the bodies of patiens with CKD due to inadequate clearance. These substances include creatinine, urea, peptides, parathyroid hormones, and protein-binding toxins (25). Research indicates that the accumulation of these toxins can directly activate immune cells, such as macrophages and lymphocytes, leading to the release of inflammatory factors and resulting in a state of micro-inflammatory (26). Additionally, accumulated toxins can adversely affect intestinal epithelial cells, disrupting their barrier function. Under physiological conditions, intestinal microorganisms form biofilms on the surface of the intestinal mucosa, which play a critical role in preventing foreign pathogens, toxins, and harmful metabolites from entering the body (27). When the intestinal barrier function is compromised, intestinal bacteria and other pathogens can translocate into the circulatory system, activate the immune system, and subsequently trigger a systemic inflammatory response (28).

### 2.2 Oxidative stress enhancement and endothelial dysfunction

Oxidative stress refers to the disruption of the balance between oxidants and antioxidants in the body, resulting in the excessive accumulation of reactive oxygen species (ROS). This accumulation can lead to the oxidation and damage of cells and tissues (29). In patients with CKD, the buildup of toxins can increase the activity of oxidants, such as nicotinamide adenine dinucleotide phosphate oxidase (30). Coupled with dietary restrictions, diuretic use, and inadequate protein energy intake, the production of antioxidants becomes insufficient (31). Resulting in the impairment of the antioxidant defense mechanism, which is characterized by a continuous increase in ROS levels within the body. Excessive ROS can not only directly damage cellula structures, but also has a double effect through the activation of the nuclear factor kappa B (NF-kB) signaling pathway (32): on the one hand, it promotes the expression of inflammatory genes and aggravates the micro-inflammatory state; on the other, increases the expression of adhesion molecules of endothelial cells, further promotes the adhesion and migration of white blood cells, thereby, disrupting endothelial barrier function. Damaged endothelial cells can release more pro-inflammatory factors and chemokines, which continuously aggravate endothelial damage and promote the development of inflammatory response (33).

### 2.3 Immune imbalance and metabolic acidosis

The kidneys are primarily responsible for clearing circulating cytokines and bacterial antigens, which plays a crucial role in maintaining immune system homeostasis (34). When renal function is impaired, this clearance capability diminishes, resulting in an imbalance of immune homeostasis. Major manifestations include increased levels of pro-inflammatory cytokines, acute phase proteins such as pentraxins, as well as dysfunctional phagocytes, T cells, and B cells. This dysfunction can trigger an autoimmune response that leads to a persistent micro-inflammatory state (35).

Additionally, with the gradual decline of renal function, the body’s acid–base balance regulation mechanism is also damaged, resulting in the weakening of the ability of renal tubular epithelial cells to secrete hydrogen ions. This change encourages the accumulation of acidic substances in the body, which eventually leads to metabolic acidosis. Research indicates that macrophages accelerate the production of TNF-*α* in acidic environments, suggesting that acidosis may further stimulate inflammation in the body (36). However, the specific mechanisms linking acidosis and inflammation remain to be fully elucidated.

### 2.4 Others

**Figure 1:**
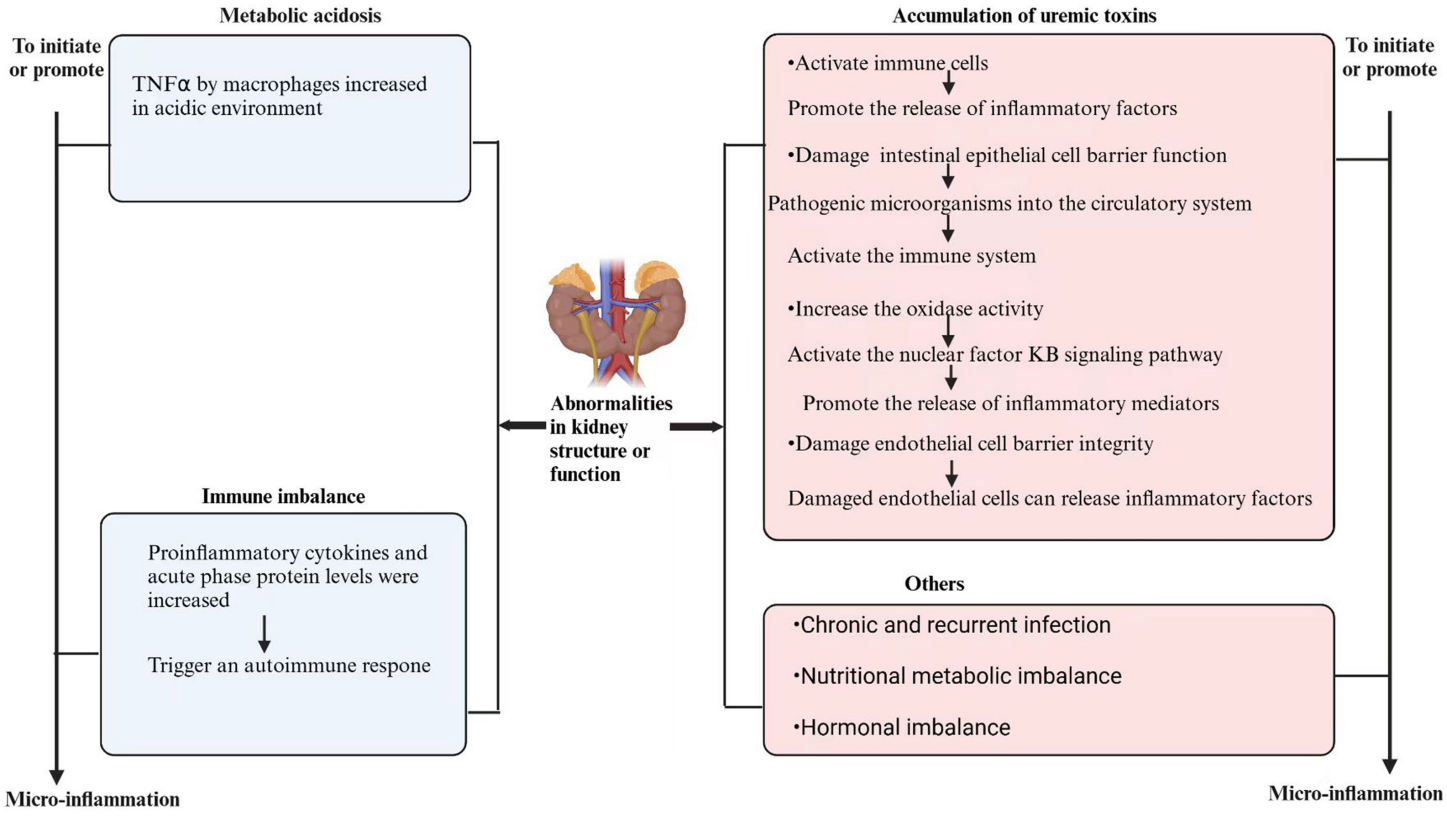
Mechanism of micro-inflamation in CKD.

Other factors include chronic and recurrent infections, abnormal nutritional metabolism, and hormone imbalancees (37). These factors collectively contribute to the micro-inflammatory state of CKD patients (Figure 1), which in turn accelerates the decline in renal function and the emergence of related complications (38), thereby creating a vicious cycle. Consequently, micro-inflammation plays a crucial role in the progression of CKD.

## 3 Relationship between NLR, PLR, SII, and CKD

### 3.1 Assessment of micro-inflammatory status in patients with CKD

Novel inflammatory markers, such as NLR, PLR and SII, have garnered significant interest due to their low cost, accessibility, and non-invasive nature (11). Recent studies have demonstrated (16, 17) that, the levels of NLR and PLR in CKD patients, are higher than those in healthy individuals and show a significant positive correlation with inflammatory markers such as CRP and IL-6. Receiver operating characteristic (ROC) curve analysis revealed that for dialysis patients, when NLR and PLR were 2.82 and 122.2, the area under the curve (AUC) to predict micro-inflammation were 0.542 and 0.610, respectively. By contrast, in non-dialysis CKD patients, when NLR and PLR were 1.98 and 116.07, the corresponding AUC values were 0.64 and 0.71, respectively. These results indicate that PLR demonstrating superior capability in identifying inflammation. However, a similar study in China showed that NLR (AUC 0.69) showed greater diagnostic value than PLR (AUC 0.55) in assessing inflammation in patients with CKD (39). When the body undergoes an inflammatory response, the number of neutrophils increases significantly and performs phagocytic function through chemotactic migration. At the same time, the apoptosis of lymphocytes increased in the inflammatory environment, while platelets were activated under inflammatory stimulation and combined with white blood cells to participate in the body’s inflammation and immune response (40). Therefore, in the inflammatory state, both NLR and PLR will increase correspondingly, thus providing a valuable reference for evaluating the micro-inflammatory state of patients with CKD.

Existing studies have demonstrated that novel inflammatory indicators, specifically NLR and PLR, serve as micro-inflammatory markers in patients with CKD. However, there is still controversy about which indicator is more sensitive to identify inflammation, and large-scale prospective studies are needed to further demonstrate. Moreover, the critical values for NLR and PLR vary significantly across different studies. This variation can be attributed, in part, to the differing stages of CKD among the included patients, as the micro-inflammatory state in dialysis patients is markedly heightened, resulting in elevated overall inflammatory indicators and consequently higher critical values. Additionally, racial differences in inflammatory indicators (41) contribute to the variability in critical values across different racial groups. Future studies should encompass multi-ethnic populations and establish the optimal thresholds for identifying inflammation in diverse demographic groups.

### 3.2 As a marker of vascular calcification in patients with CKD

Vascular calcification (VC) refers to the multifactorial processes that lead to the deposition of calcium-phosphate complexes within the vascular system, which is recognized as a significant risk factors for the onset and mortality of cardiovascular diseases (CVD) (42). Consequently, the early identification and prevention of VC progression can contribute to the timely prevention of CVD in patients with CKD. Studies have indicated that inflammatory cytokines play a mediating role in the development of VC in CKD patients (43), with elevated levels of IL-6 observed in VC patients who do not exhibit symptoms of infection (44). This suggests that inflammatory markers may be associated with VC. A cross-sectional study by Roumeliotis et al. (45), which included 158 CKD patients supported this hypothesis. This study found a significant positive correlation between NLR and dephosphorylated non-carboxylated Gla protein (dp-ucMGP) in CKD patients (*r* = 0.43, *p* < 0.0001). Regression analysis, adjusted for multiple risk factors related to NLR, indicated that dp-ucMGP is an independent predictor of NLR (*p* < 0.0001). Dp-ucMGP, an inactive form of the VC inhibitor MGP, has been demonstrated in several studies to be a reliable marker of VC and to predict the morbidity and mortality associated with CVD (41, 46). Therefore, NLR may also serve as a marker for VC in CKD patients. Studies conducted by Li (47) and Chandra’s team (48) in both non-dialysis and dialysis patients further validated this finding, indicating that PLR predicted VC in dialysis patients more accurately than NLR (AUC was 0.705 and 0.671, respectively). Currently, VC is generally regarded as a chronic inflammatory lesion involving various inflammatory cells, factors and adhesion molecules (49). Additionally, due to various factors, patients with CKD experience excessive platelet activation. Activated platelets not only increase the release of inflammatory factors, enhancing the interaction between vascular smooth muscle cells (VSMC) and these factors, but also lead to the transformation of VSMC from a contractile phenotype to a synthetic phenotype, thereby promoting cell migration and proliferation, and contributing to the remodeling of the extracellular matrix, ultimately facilitating the occurrence of VC (50).

Existing studies have demonstrated that monitoring NLR and PLR can aid in predicting the occurrence of VC in CKD patients, thereby facilitating the early identification of high-risk groups for CVD in clinical practice. However, the optimal predictive value of these ratios necessitates further validation through large-scale prospective studies.

### 3.3 Assessing the nutritional status of patients with CKD

Malnutrition is more prevalent among patients with CKD, primarily manifesting as reduced protein and energy reserves, decreased muscle mass, and a heightened catabolic state, commonly referred to as protein-energy wasting (PEW) (51). The severity of PEW escalates with the progression of CKD and is linked to adverse clinical outcomes in these patients (52, 53). Chronic micro-inflammatory are recognized as one of the primary contributors to malnutrition in CKD patients, resulting in PEW by promoting muscle catabolism, triggering anorexia, reducing insulin-like growth factor-1 (IGF-1) secretion, and decreasing activity levels (54). Given the proposed relationship between inflammation and PEW, the predictive value of inflammatory markers for malnutrition in CKD has garnered significant interest among researchers. Han et al. (55) observed in non-dialysis CKD patients, the incidence of PEW increased by 1.393 times for each one-unit increase in NLR [95% *CI* (1.078–1.800), *p* = 0.011]. As for hemodialysis patients, study by Hu et al. (56), indicated that high PLR was an independent risk factor for PEW, and the risk of PEW in the Q4 (highest quartile) group was 2.93 times that of the Q1 (lowest quartile) group [95% *CI* (1.50–5.73), *p* = 0.002]. When PLR was 144.09 predicted PEW sensitivity and specificity for hemodialysis patients were 61 and 58%, respectively. Ran et al. (57) identified SII as another independent risk factor for PEW in hemodialysis patients. The incidence of PEW was higher when SII was 520 (AUC 0.725), and the sensitivity and specificity were 69 and 70%, respectively. While platelets are primarily recognized for their roles in hemostasis and thrombosis, several studies (58, 59) have shown that they also participate in the body’s inflammatory processes. Activated platelets can release various pro-inflammatory factors, such as CXC chemokine ligand 1, IL-8, and TNF-*β* (60). These inflammatory cytokines can stimulate the hypothalamic satiating center, leading to loss of appetite, delayed gastric emptying, and increased muscle catabolism (61). Furthermore, some researchers have found that pro-inflammatory cytokines can directly act on L cells in the small intestine, resulting in increased secretion of glucagon-like peptide-1 (GLP-1), which leads to decreased food intake and weight loss (62). Collectively, these factors can ultimately contribute to the development of PEW.

Existing studies have demonstrated that PLR and SII possess certain predictive value regarding PEW in dialysis patients, while NLR is relevant for non-dialysis patients with CKD. However, it is limited by low insensitivity and specificity, future research is anticipated to explore whether the combined use of multiple indicators can enhance predictive efficiency. Furthermore, there is currently no conclusive research on whether PLR and SII provide equivalent predictive value for non-dialysis patients with CKD, nor on whether NLR holds the same predictive value for dialysis patients. Additionally, the precise efficacy of nutritional supplements combined with targeted anti-inflammatory interventions on PEW remains to be thoroughly investigated.

### 3.4 Predicting the dialysis access failure

Autologous arteriovenous fistula (AVF) is the preferred and primary vascular access for patients undergoing hemodialysis, often referred to as the lifeline for these individuals (63). However, due to the repeated puncture of hemodialysis needles and the sustained damage associated with the hemodialysis process (64), AVFs are susceptible to various complications, with stenosis and thrombosis being the most prevalent (65). Although the precise mechanisms underlying these complications remain unclear, researches have indicated (66, 67) that prolonged chronic inflammatory stimulation may lead to intimal hyperplasia and thrombosis, which could be among the key factors contributing to AVF dysfunction. Retrospective analyses by Pasqui et al. (68) and Kaller et al. (69) have shown that in patients with end-stage renal disease (ESRD) patients who received their first AVF creation, preoperative elevated levers of NLR, PLR, and SII are associated with AVF failure. Multivariate Cox regression analysis confirmed that a high preoperative NLR is an independent risk factor for AVF failure including stenosis and thrombosis, with an odds ratio (OR) of 2.53[95% *CI* (1.89–3.11)]. Moreover, when NLR exceeded 4.21, the likelihood of AVF failure significantly increase [AUC 0.7733, 95% *CI* (0.7128–0.8339), *p* < 0.0001]. Further study has suggested that monitoring PLR may be beneficial in predicting restenosis of AVF within 12 months following successful percutaneous transluminal angioplasty (PTA) (70). A large prospective study by Ren et al. (71), which included 2,690 hemodialysis patients, revealing that patients undergoing AVF for the first time exhibited the lowest risk of channel failure. Elevated systemic inflammatory markers, such as NLR, PLR, and SII, along with a previous history of PTA surgery, were found to independently predict hemodialysis channel failure. Notably, SII demonstrated the highest predictive value. Prediction models based on SII was established, with a concordance index (c-index) of 0.6314 [95% *CI* (0.6249–0.6589)] for predicting 6-month access survival and 0.6441 [95% *CI* (0.6212–0.6670)] for 12-month access survival. However, the relatively low c-indexes indicate that the predictive power of any single marker for dialysis access failure is limited. It is also worth noting that vascular endothelial cells play a critical role in AVF maturation through the secretion of vasodilatory factors and a series of pro-inflammatory molecules (72). Conversely, the micro-inflammatory state associated with ESRD may lead to endothelial cell dysfunction, potentially affecting the maturation of AVF. This may partially elucidate the association between inflammatory markers and AVF dysfunction.

Existing studies have demonstrated that monitoring relevant indicators can facilitate the observation of adverse outcomes related to AVFs. Patients with a history of pathway dysfunction or previous PTA surgery, along with elevated systemic inflammatory markers, should be considered high-risk groups for short-term (12 months) dialysis pathway failure, warranting closer monitoring. This monitoring should include vascular ultrasound and blood-related indicators. Given the critical role of AVFs in hemodialysis, subsequent studies are expected to further elucidate whether interventions targeting factors associated with pathway failure, such as inflammation, can effectively reduce the incidence of adverse AVF outcomes.

### 3.5 Predicting EPO resistance

Due to erythropoietin (EPO) deficiency, iron metabolism disorders, chronic inflammation, and other factors, most patients with CKD experience varying degrees of anemia, commonly referred to as renal anemia (73). The severity of renal anemia is closely linked to the quality of life and prognosis of CKD patients. The primary cause of renal anemia is the relative insufficiency of EPO secretion, which makes EPO supplementation the standard treatment for this condition. However, some patients exhibit poor therapeutic responses to EPO, a phenomenon known as EPO resistance, where in the hemoglobin levels of patients fail to reach the target range despite adequate EPO treatment (74). Relevant studies indicate that micro-inflammation and oxidative stress are the main causes of EPO resistance in CKD patients (75), and it has been found that those with higher levels of NLR and PLR have a higher risk of EPO resistance in dialysis (76). A multicenter cross-sectional study in Spain (77) demonstrated that NLR (standardized *β* = 0.173, *p* < 0.0001, *r*^2^ = 0.029) and PLR (standardized *β* = 0.32, *r*^2^ = 0.103, *p* < 0.0001) can predict the erythropoietin response index (logERI), which is defined as the resistance index of erythropoiesis-stimulating agents (ESAs). The ERI is calculated by dividing the weekly dose of EPO (in IU) by the patient’s dry weight (in kg) and then dividing by the hemoglobin concentration (in g/dL). ERI is regarded as a reliable marker of erythropoietin resistance, with PLR demonstrating superior predictive capability compared to NLR. Contrasting opinions were presented in a similar study conducted in China (78), which identified only NLR as a significant predictor of log erythropoietin resistance index (logERI) (standardized *β* = 0.13, *r*^2^ = 0.039, *p* = 0.024), while PLR did not reach significance (*p* = 0.063). When the body stimulates the release of cortisol in a state of stress, this can lead to a reduction in lymphocyte counts, thereby impacting immune function and potentially diminishing the EPO (75), which may be one of the mechanisms of high NLR and PLR leading to EPO resistance.

Existing studies indicate that the straightforward calculation of NLR and PLR may offer a preliminary framework for clinicians to identify EPO resistance. However, the low *r*^2^ values reported in these studies suggest that the correlation is insufficient, rendering these ratios inadequate as independent predictors of EPO resistance.

### 3.6 Predicting depression in ESRD patients

Depression is the most prevalent mental illness among patients with CKD, particularly in those with ESRD, and poses a serious, often life-threatening risk (79). Research indicates that depression not only increases the frequency and duration of hospitalizations in CKD patients but also elevates the risk of cardiovascular events, suicide rates, and mortality associated with CKD (80–82). A significant challenge in the clinical management of depression among dialysis patients is the delayed recognition of depressive symptoms and the subsequent diagnosis of the condition (83). Currently, commonly employed diagnostic methods for depression include clinical interviews, self-rating scales, various rating scales, and psychological tests (84). However, these methods can be influenced by subjective factors from healthcare providers and the cognitive abilities of patients, which may lead to missed or incorrect diagnoses. Thus, the utilization of objective indicators to aid in the diagnosis of depression is crucial. It has been established that vitamin D deficiency and thyroid dysfunction contribute to an increased risk of depression in CKD patients (85, 86), and the assessment of relevant biomarkers can facilitate the diagnosis of depression. NLR, a simple and readily accessible inflammatory marker, has been associated with various depressive states, including post-stroke depression (87), adolescent depression (88), and major depression (89). Feng et al. (90) recently grouped 160 MHD patients by Patient Health Questionnaire-9 (PHQ-9) score and found that high NLR was an independent risk factor for moderate to severe depressive symptoms [*OR* 1.441, 95% *CI* (1.017–2.042), *p* = 0.04]. This is consistent with the conclusion of another study that utilized the Baker Depression Scale score to diagnose depression in dialysis patients (91), suggesting that monitoring NLR may facilitate the early identification and diagnosis of depressive symptoms in this population. Additional studies have indicated that levels of inflammatory cytokines, such as CRP, IL-6, and TNF-*α*, are elevated in patients with depression compared to non-depressed individuals. Furthermore, the increase in these inflammatory cytokines correlates with the severity of depression (92, 93). The NLR is regarded as a significant marker of inflammation and immune system activation; a higher ratio indicates a greater degree of inflammation and immune response. Concurrently, the secretion of pro-inflammatory cytokines also increases (91). However pro-inflammatory cytokines can reduce the conversion of tryptophan to 5-hydroxytryptamine (5-HT) while simultaneously promoting its degradation to kynurenine through the activation of indoleamine-2, 3-dioxygenase (94). The reduction in 5-HT levels is recognized as a crucial factor in the pathogenesis of depression (95). Furthermore, the elevated production of kynurenine results in an increased synthesis of its metabolite, quinolinic acid, which is believed to contribute to the neurotoxicity associated with the development of depression (96). Psychosocial stress is another significant contributor to depression, and the inflammation triggered by prolonged psychological stress is closely linked to the onset of depressive symptoms (97). Thus, inflammation may interact with psychological factors to facilitate the emergence of depressive symptoms through a complex interplay of pathophysiological and behavioral mechanisms (98).

Existing studies indicate that dialysis patients exhibiting elevated levels of inflammatory markers are at an increased risk of depression. Furthermore, monitoring NLR may prove beneficial in predicting various forms of depression. However, research regarding PLR, SII, and their relationship with depression in dialysis patients remains insufficient.

### 3.7 Prediction of proteinuria

Proteinuria is a hallmark of kidney damage and is strongly associated with the progression of CKD (98). Early detection and management of proteinuria are critical to prevent further kidney injury. Current clinical methods for estimating proteinuria, such as the urinary total protein-to-creatinine ratio (UPCR), urinary albumin-to-creatinine ratio (UACR), and 24-h urinary protein excretion, are susceptible to influence from various environmental factors and patient behaviors, which compromises their accuracy and reliability (99). Therefore, there is a pressing need to identify new biomarkers for proteinuria. Aneez et al. (100) conducted a cross-sectional study involving 85 patients with stage CKD stages 2–4 and found that both NLR and PLR were significantly positively correlated with proteinuria in this cohort. Similarly, Tutan et al. (101) observed that among 327 patients with uncontrolled diabetes, the median NLR in the proteinuria group was significantly higher than in the non-proteinuria group (*p* < 0.001). Multiple logistic regression analysis, demonstrated that NLR is an independent predictor of proteinuria [Exp(B) = 1.606, 95% *CI* (1.208–2.136), *p* = 0.001]. Specifically, the risk of developing proteinuria increased 1.93-fold when NLR exceeded 1.93. These findings suggest that NLR may serve as a valuable predictor of proteinuria in patients with moderate to advanced CKD and could aid in the early diagnosis of diabetic nephropathy. Regarding the potential mechanisms underlying the association between NLR and proteinuria, immune dysfunction characterized by altered neutrophil and lymphocyte counts may impair the immune response to kidney injury, thereby facilitating the development of proteinuria (102).

Existing studies have demonstrated that NLR and PLR can serve as indicators of albuminuria severity in patients with moderate to advanced CKD. Additionally, monitoring these markers could provide an additional tool for predicting and managing proteinuria in CKD patients. However, research investigating the correlation between SII and proteinuria is currently insufficient.

## 4 Prognosis of NLR, PLR, SII, and CKD

NLR, PLR and SII have been extensively studied in the fields of malignant tumors, cardiovascular or cerebrovascular diseases and respiratory diseases (12–15). With regard to the renal field, The value of these indicators in the prognosis of patients with CKD is also gradually being emphasized.

### 4.1 Predicting cardiovascular events

CVD is the most prevalent complication and leading cause of death among patients with CKD, with an average incidence that is 15 to 20 times higher than that of the general population. Furthermore, the mortality rate attributed to CVD accounts for approximately 40 to 50% of deaths in patients with ESRD (103). However, traditional risk factors—including obesity, hypertension, diabetes, and smoking-do not fully account for the elevated incidence and mortality of CVD in CKD patients. Relevant studies indicate that micro-inflammation plays a crucial role in the onset and progression of CVD in this population (112). For instance, Zhu et al. (18) followed up 555 hemodialysis patients for 24 months and observed that NLR and PLR were significantly higher in the cohort with CVD compared to those without. Additionally, high NLR was accompanied by higher markers of myocardial injury as, cardiac troponin I (cTnI), and creation kinase-MB (CK-MB), suggesting that a high NLR may have a certain predictive value of myocardial injury. The AUC for CVD prediction using NLR was 0.84, with sensitivity and specificity values of 75 and 79%, respectively. Chen et al. (19) found in consecutive ambulatory peritoneal dialysis (CAPD) patients that the risk of CVD in the high PLR group was 1.05 times higher than that in the low PLR group [95% *CI* (1.03–1.07), *p* < 0.01], and that the risk of CVD in CAPD patients was higher when the PLR was ≥118.35, with an AUC of 0.92 [95% *CI* (0.85–0.98), *p* < 0.01]. In another study (20), high SII upon admission was identified as an independent risk factor for CVD mortality in patients with CKD combined with acute coronary syndrome (ACS), with a hazard ratio (*HR*) of 1.865 [95% *CI* (1.197–2.907), *p* = 0.006]. Furthermore, the incorporation of SII into existing prognostic risk prediction models for CKD significantly enhanced their predictive performance. Neutrophils are implicated in vascular endothelial damage and atherosclerosis, while activated platelets are considered a crucial component of atherosclerosis, which is fundamental to the development of CVD (104). Therefore, when NLR, PLR and SII are elevated, they are often associated with a higher incidence of cardiovascular events, indicating that these indicators are of great value in predicting cardiovascular complications in patients with CKD.

Existing studies have demonstrated that NLR, PLR, and SII possess predictive value regarding the occurrence, progression, and prognosis of CVD in patients with CKD. However, further research is required to establish the optimal sensitivity values for these predictive markers. Additionally, it is essential to explore anti-inflammatory pathways as potential therapeutic targets to mitigate CVD morbidity and mortality.

### 4.2 Predicting kidney outcomes

Diabetes mellitus, hypertension, and obesity are the primary etiologies of CKD, which often progresses to ESRD even when these underlying conditions are managed. This progression occurs because the loss of renal units leads to hypertrophy and increased ultrafiltration in the remaining renal units (105). ESRD necessitates renal replacement therapy, either through dialysis or renal transplantation, which not only diminishes the quality of life for patients but also imposes a significant medical and economic burden globally. However, predicting the progression of CKD presents considerable challenges in clinical practice. Consequently, identifying suitable prognostic monitoring indicators is essential for developing effective prevention and management strategies. In a prospective study by Yoshitomi et al. (106), found a significant negative correlation between high NLR and estimated glomerular filtration rate (eGFR) (*β* = −0.23, *p* < 0.01) in non-dialysis patients. Additionally, patients in the high NLR group exhibited a 1.67-fold increased risk of entrying into dialysis, compared to those in the low NLR group [95% *CI* (1.02–2.77)]. This study established a foundation for predicting renal outcomes in CKD using NLR. Subsequent studies by Lan et al. (105) and Rashi et al. (107) further corroborated these findings, indicating that a higher NLR is associated with a more rapid decline in eGFR. They also noted that NLR independently predicts this decline, demonstrating superior predictive ability compared to other indicators such as PLR and SII. Neutrophils can release chemotactic substances that enhance their migration to the kidney, exacerbating glomerular injury, which contributes to the worsening of renal function in CKD (105). Consequently, elevated NLR is also associated with aggravated kidney damage.

Existing studies have demonstrated that NLR, PLR, and SII can independently predict the rapid decline of eGFR. Monitoring the dynamic changes in these indicators may facilitate the observation of renal function progression trends, thereby enabling early targeted interventions that are anticipated to reduce the incidence of adverse renal outcomes.

### 4.3 Predicting infection events

Infections are recognized as the second leading cause of death among patients with ESRD (108). Compared to the general population, patients with CKD face a markedly elevated risk of developing infections due to factors such as immune dysfunction, comorbidities, and treatment-related issues, including the use of immunosuppressants and dialysis. Among the most prevalent infectious diseases encountered in these patients are pulmonary infections, catheter-associated bloodstream infections, and peritonitis. Infections not only impose a substantial burden on the organism and can be fatal in severe cases, but available data also suggest that the occurrence of infections is associated with an increased risk of subsequent CVD (113). Consequently, early recognition of infections and timely, effective treatment are crucial for improving the poor prognosis often linked with these complications. A retrospective cohort study (108) involving 100 patients undergoing MHD found that NLR is a valid predictor of lung infections, with an increased risk of lung infection observed when NLR exceeds 5.52 [AUC 0.829, 95% *CI* (0.740–0.897)]. Yan et al. (109) demonstrated that NLR serves as an independent risk factor for pneumonia in hemodialysis (HD) patients, with each 1-unit increase in NLR correlating to a 7.2% increase in the risk of developing pneumonia (*p* = 0.035). Another study (110) indicated that an NLR of ≥4.485 holds diagnostic value for catheter-related bloodstream infections (CRBSI) in HD patients. In patients undergoing peritoneal dialysis (PD), peritonitis is the most common complication, frequently referred to as PD-associated peritonitis (PDAP) (114). Relevant studies have noted that the likelihood of treatment failure for PDAP increases with an NLR > 6.53 [*OR* 1.82, 95% *CI* (1.05–3.15), *p* < 0.05]. Compared to patients with an NLR < 3.75, those with an NLR > 6.53 face a 3.41-fold increased risk of treatment failure (111). These findings suggest that monitoring NLR can serve as an early warning for the onset of PDAP and assist in evaluating its therapeutic efficacy. Elevated inflammatory markers indicate a heightened inflammatory response within the body, leading to increased susceptibility to infections. Therefore, a higher NLR correlates with an increased likelihood of lung infections, catheter-related bloodstream infections, and peritonitis.

Existing studies have demonstrated that NLR possesses diagnostic value for lung infections and catheter-associated bloodstream infections in patients undergoing HD, and it can also be utilized to evaluate the therapeutic effect of PDAP. However, there is a paucity of research concerning the prognostic value of PLR, SII, and their correlation with infections in CKD. NLR, being more cost-effective and easier to obtain than traditional inflammatory markers, is anticipated to serve as a valuable biomarker for assessing infectious diseases associated with CKD complications.

### 4.4 Predicting all-cause mortality

CKD is associated with nearly 10 million deaths annually (3). Therefore, in addition to effective management of CKD comorbidities and related complications, screening and managing patients at higher risk of mortality is a critical step in reducing overall mortality rates. Elevated NLR and PLR have been correlated with reduced survival in patients with advanced rectal cancer undergoing immunotherapy (12) and with an increased risk of all-cause mortality in patients with chronic obstructive pulmonary disease (15). Recently, several retrospective cohort studies involving patients with ESRD (21–23) have demonstrated that all-cause mortality in this population increases with higher levels of NLR and PLR, and noted that NLR was independently associated with all-cause mortality, while PLR did not. Among these studies, Woziwodzka et al. (21) reported that an NLR ≥ 3.9 was a significant predictor of 5-year all-cause mortality in ESRD patients [HR 2.23, 95% *CI* (1.10–4.50), *p* = 0.025]. Furthermore, for every 1-unit increase in NLR, the risk of death from all causes within 5 years increased by 1.26-fold [95% *CI* (1.06–1.51), *p* = 0.009]. Mayne et al. (22) found that hemodialysis patients with an NLR ≥ 8.23 had a 63% increased risk of all-cause mortality compared to those with an NLR < 3.12 [*HR* 1.63, 95% *CI* (1.32–2.00)]. Zhang et al. (23) followed up 360 HD patients with excluded history of cardiovascular disease for 71 months, and also found that high NLR was closely associated with all-cause mortality, while affirming the value of high PLR in predicting cardiovascular mortality in HD patients. Elevated NLR and PLR often reflect an increased inflammatory state in the body. Inflammation promotes the progression of atherosclerosis and leads to coronary artery damage, thus exacerbating the occurrence of myocardial ischemia, aggravating the original cardiovascular diseases, and leading to increased mortality (111). At the same time, elevated NLR and PLR means a relative proportion of lymphocytes decreased or neutrophils and platelets increased. The decreased number of lymphocytes may impair the body’s ability to fight infection and affect the adaptive immune response. Excess neutrophils and platelets may lead to an overactive innate immune response, resulting in tissue damage (40). All these suggest that a higher level of inflammatory indicators reflects a poor prognosis.

Existing studies have established that the predictive value of NLR for all-cause mortality in hemodialysis patients is increasingly acknowledged. Monitoring NLR can aid in identifying individuals at high risk for all-cause mortality; therefore, such high-risk patients should be closely monitored and receive targeted therapies to mitigate all-cause mortality.

## 5 Summary and outlook

Chronic micro-inflammatory status is considered a hallmark of CKD patients, which not only participates in disease progression, but also plays an important role in the development of multiple complications and overall prognosis of patients, therefore, monitoring and controlling chronic inflammation is an important step in the management of CKD. Novel inflammation markers are easier to obtain and more cost-effective than traditional inflammation markers, so it is worthwhile to further explore their potential applications in future studies. Available studies have found that PLR is superior to NLR in predicting the occurrence of VC and cardiovascular mortality of CKD patients, however, it is still controversial in predicting EPO resistance and all-cause mortality. NLR is more recognized in predicting EPO resistance, rapid progression of renal function, and infection-related complications. Higher NLR in patients with CKD may be regarded as the occurrence of depression. The results of this study are summarized in the following table, AVF failure and all-cause mortality, close monitoring and treatment of such patients is expected to reduce the incidence of related complications and poor prognosis. There is a lack of research on the relationship between PLR, SII and depression or infection-related diseases in CKD patients, as well as the relationship between SII and CKD-related complications and prognosis.

Current studies have primarily concentrated on the short-term prognosis of patients with intermediate and advanced CKD. However, the impact of inflammatory markers on the long-term prognosis of CKD in its early stages remains unclear. Additionally, despite the valuable insights provided by existing studies, a notable limitation of the current body of research is the relative paucity of large-scale studies that adequately represent diverse populations. The majority of the available literature tends to focus on single-center or multi-center studies with limited sample sizes, which may not fully capture the variability inherent in different ethnic, cultural, and socioeconomic groups. This limitation restricts the generalizability of the findings and can impact the applicability of conclusions drawn from these studies to broader populations. Furthermore, markers such as NLR, PLR, and SII are dynamic and may be affected by body stress, autoimmune and hematological diseases, some drugs, and possible measurement errors. Consequently, results derived from a single measurement may not accurately represent the inflammatory status of CKD patients at that time. While retrospective studies provide foundational data, they typically rely on single measurements. Future cohort studies are anticipated to explore whether multiple measurements are necessary to achieve a more accurate average value in research concerning the relationship between inflammatory indices and CKD.

Future studies should concentrate on evaluating the predictive value of inflammatory markers for long-term prognosis in patients with early-stage CKD, as well as assessing the impact of interventions aimed at mitigating chronic inflammation on the overall prognosis of these patients. To validate current findings and better define the optimal range of predictive values for these markers across diverse populations, large multicenter, ethnically diverse, prospective studies are essential. Concurrently, it is important to further investigate the application of novel inflammatory markers at various stages of CKD, with the goal of optimizing diagnostic and therapeutic strategies for CKD patients.
